# Intestinal microbial remodeling and metabolite regulation may be related to the improvement of social avoidance in depressed mice by Modified Xiaoyaosan

**DOI:** 10.3389/fmicb.2026.1774975

**Published:** 2026-07-27

**Authors:** Ting Linghu, Ting Hu, Zhenning Wu, Qi Wang, Yunhao Zhao, Yujing Wang, Kaiwen Li, Xuemei Qin, Junsheng Tian, Ruiping Zhang

**Affiliations:** 1 The Radiology Department of Shanxi Provincial People’s Hospital Affiliated to Shanxi Medical University, Taiyuan, Shanxi, China; 2 Institute of Medical Technology, Shanxi Medical University, Taiyuan, Shanxi, China; 3 Modern Research Center for Traditional Chinese Medicine, Shanxi University, Taiyuan, Shanxi, China

**Keywords:** depression, intestinal permeability, multi-omics integration analysis, social avoidance behavior, traditional Chinese medicine

## Abstract

**Background:**

Increased intestinal permeability is implicated in the pathogenesis of depression. Modified Xiaoyaosan (MXYS) has demonstrated antidepressant-like effects in chronic social defeat stress (CSDS) mice, particularly in improving social avoidance behavior. However, direct evidence that MXYS improves depressive behavior by regulating intestinal permeability remains lacking.

**Methods:**

Using a CSDS-induced depression mouse model, we investigated the effects of MXYS through an integrated multi-omics approach, combining intestinal histopathology, microbiome, metabolomics, and molecular biology techniques. This strategy allowed systematic assessment of pathological changes, gut microbial composition, metabolite profiles, and protein expression.

**Results:**

MXYS treatment significantly alleviated social avoidance behavior in CSDS mice. It upregulated the expression of tight junction proteins in the colon, jejunum, and hippocampus. MXYS also reduced serum permeability markers of lipopolysaccharide (LPS), D-lactate (D-Lac), and diamine oxidase (DAO), as well as concentrations of pro-inflammatory cytokines TNF-α and IL-1β in both intestinal tissues and the hippocampus. Gut microbiota analysis revealed that MXYS notably decreased the relative abundance of *Desulfovibrio* in the colon, which was associated with modulation of β-alanine metabolism. In the jejunum, MXYS increased the relative abundance of *Clostridium sp. mbf VZ-132* and influenced glycerophospholipid metabolism.

**Conclusion:**

MXYS alleviates social avoidance in CSDS-induced depressed mice is associated with the restoration of intestinal barrier function via gut microbiota-mediated pathways. Specifically, it may reduces colon *Desulfovibrio* abundance to modulate β-alanine/aspartate metabolism and enhance claudin-1 expression, while increasing jejunal *Clostridium sp. mbf VZ-132* to regulate glycerophospholipid metabolism and upregulate occludin expression. These findings suggested that MXYS may improves depression-like behavior through segment-specific microbial and metabolic modulation of intestinal permeability.

## Graphical Abstract

**Figure:**
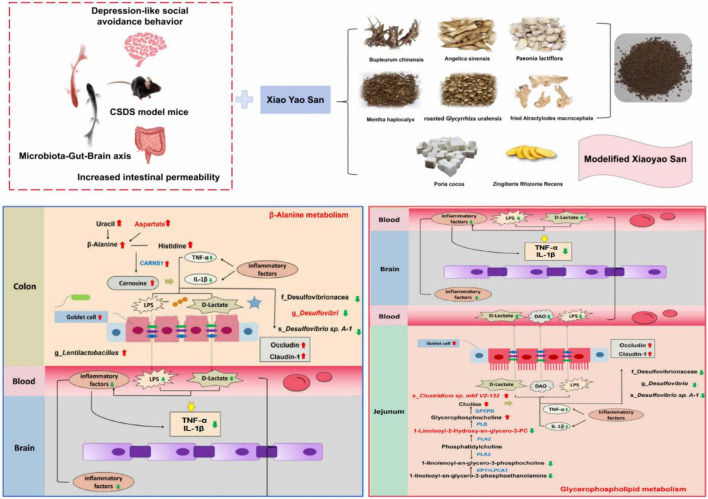
Schematic illustration of the proposed mechanisms by which Modified Xiaoyaosan (MXYS) ameliorates social avoidance behavior in chronic social defeat stress (CSDS) mice through segment-specific remodeling of gut microbiota and metabolites in the colon and jejunum, leading to restoration of intestinal barrier integrity and suppression of neuroinflammation.

## 1 Introduction

Depression, a highly debilitating mental health disorder, has become a critical global public health issue, accounting for the greatest disease burden among psychiatric conditions (Karimi et al., 2025). According to the World Health Organization, more than 350 million people worldwide suffer from depression, and it is projected to become the leading cause of disease burden by 2030 (Wang Q. et al., 2023; Richardson et al., 2025). Notably, approximately 50%–75% of individuals with depression exhibit significant social avoidance behavior, a core symptom that severely impairs social functioning and quality of life (Jin Y. et al., 2025). However, the underlying mechanisms of social avoidance behavior in depression remain poorly understood, and few studies have specifically focused on this behavioral dimension. In recent years, the microbiota-gut-brain axis (MGBA) has gained recognition as a pivotal pathway implicated in the pathophysiology of depression, offering a novel insight for investigating behavioral symptomatology (Bandelow et al., 2023).

Conventional pharmacological interventions for social avoidance behavior, such as selective serotonin reuptake inhibitors, although effective, are often associated with long-term adverse effects including gastrointestinal discomfort, which may compromise treatment adherence and efficacy (Liu et al., 2020). In contrast, traditional Chinese medicine formulas (TCMFs), characterized by multi-component nature, holistic therapeutic approach, and fewer adverse effects, have demonstrated efficacy in improving social avoidance behavior (Wang and Wu, 2020; Zhang B. et al., 2025). Xiaoyaosan, a classical TCM prescription documented in “*Tai Ping Hui Min He Ji Ju Fang*” from the Song Dynasty, is widely used to soothe the liver and relieve depressive symptoms (Kwon et al., 2024; Zhao et al., 2024). Our previous work revealed that the adverse reactions of Xiaoyaosan decoction, such as dry mouth and sensation of dryness, were suspected to be associated with *Zingiberis Rhizoma Recens*. Additionally, chemical composition attribution analysis showed that *Poria* contributed minimally to the antidepressant effect, and its spleen-strengthening function overlapped with that of *Atractylodis Macrocephalae Rhizoma*. Therefore, both *Poria* and *Zingiberis Rhizoma Recens* were removed to generate the modified formulation of Modified Xiaoyaosan (MXYS). The optimized composition comprises six medicinal herbs: *Bupleuri Radix*, *Angelicae Sinensis Radix*, *Paeoniae Radix Alba*, *fried Atractylodis Macrocephalae Rhizoma*, *roasted Glycyrrhizae Radix et Rhizoma*, and *Menthae Haplocalycis Herba* (Huang et al., 2025). Our previous studies have shown that MXYS significantly improves social avoidance behavior in chronic social defeat stress (CSDS) mice (Zhao et al., 2025). MXYS offers a comprehensive treatment alternative for patients with mild to moderate depression, exhibiting multi-target and multi-pathway mechanisms that underscore the systemic therapeutic advantages of TCMFs in managing complex neuropsychiatric conditions.

Recent multi-omics studies have demonstrated that natural products and herbal extracts can ameliorate disease phenotypes by remodeling gut microbiota and restoring metabolic homeostasis (Hao et al., 2025; Su et al., 2023; Yang et al., 2025). For instance, dietary cherry juice alleviates obesity by resolving gut microbiota dysbiosis and elevating tight junction proteins (Wang Z. et al., 2023). Similarly, *Abelmoschi corolla* polysaccharide restores immunosuppression through a coordinated axis of gut barrier restoration, microbiota remodeling, and systemic metabolic regulation (Zhang X. et al., 2025). These studies are crucial for understanding how complex Chinese herbal compounds play a multi-target role. A key mechanism linking gut dysfunction to depression involves increased intestinal permeability, which disrupts MGBA homeostasis and facilitates systemic inflammation. A seminal review emphasized that intestinal hyperpermeability contributes to brain-gut axis dysregulation and that therapeutic restoration of barrier integrity may improve symptoms of extraintestinal disorders, including depression (Grover et al., 2025). TCMFs may exert protective effects on the intestinal barrier through anti-inflammatory activity, modulation of gut microbiota, and enhancement of epithelial repair, thereby potentially ameliorating behavioral deficits such as social avoidance. This approach represents a promising integrated strategy for concurrent gut-brain intervention, though further mechanistic and clinical validation is required.

In this study, we aimed to investigate how MXYS improves social avoidance behavior in CSDS mice. Using intestinal permeability as an entry point into MGBA pathophysiology, we focused on the colon, jejunum, and hippocampus as key target tissues. By integrating behavioral assessments with multi-omics approaches, including 16S rRNA sequencing, metabolomics, and molecular biology techniques, we sought to establish a potential “marker species–potential metabolite markers–intestinal permeability” correlation network. This research provided a novel theoretical framework and a targeted MGBA-based strategy for the treatment of psychiatric disorders.

## 2 Methods and materials

### 2.1 Animals

Specific pathogen-free (SPF) male C57BL/6N mice (6–8 weeks old, 18–22 g) were used as experimental subjects. Male CD-1 retired breeder mice (8–10 months old, 35–45 g) served as resident aggressors. All animals were purchased from Beijing Charles River Animal Technology Co., Ltd (SCXK 2021-0006). Mice were housed under standard laboratory conditions (temperature: 25 °C ± 2 °C; humidity: 50% ± 10%) on a 12 h light/dark cycle, with ad libitum access to food and water. After arrival, animals were acclimatized to the facility for at least 7 days prior to experimentation.

All procedures were conducted in strict accordance with the NIH Guide for the Care and Use of Laboratory Animals and the Chinese Animal Welfare Guidelines. The study protocol was reviewed and approved by the Committee of Scientific Research at Shanxi University (Approval No. SXULL2021065).

### 2.2 Preparation and quantity control of MXYS

Modified Xiaoyaosan contains the following six medicinal herbs in a specific proportion (6: 6: 6: 6: 3: 2): Bupleuri radix (Bupleurum chinense DC.), Radix paeoniae alba (Paeonia lactiflora Pall.), Radix angelica sinensis [Angelica sinensis (Oliv.) Diels.], Rhizoma atrac-tylodes macrocephalae (Atractyles macrocephala Koidz.), Radix et rhizoma glycyrrhizae (Glycyrrhiza uralensis Fisch.), and Mentha haplocalyx heaba (Mentha-haplocalyx Briq.). All herbs were purchased from the Medicinal Materials Company of Herentang (Taiyuan, Shanxi, China) and authenticated by Professor Xuemei Qin. All herbal materials were exacted with eight times 75 % ethanol, and the residue were exacted with eight times pure water. All filtrate was mixed and condensed to 2.12 g/ml. According to our previously constructed quality control method (Gao et al., 2020), the results of fingerprint and content determination are shown in Supplementary Figure 1 and Supplementary Tables 1, 2.

### 2.3 Chronic social defeat stress model and drug administration

**FIGURE 1:**
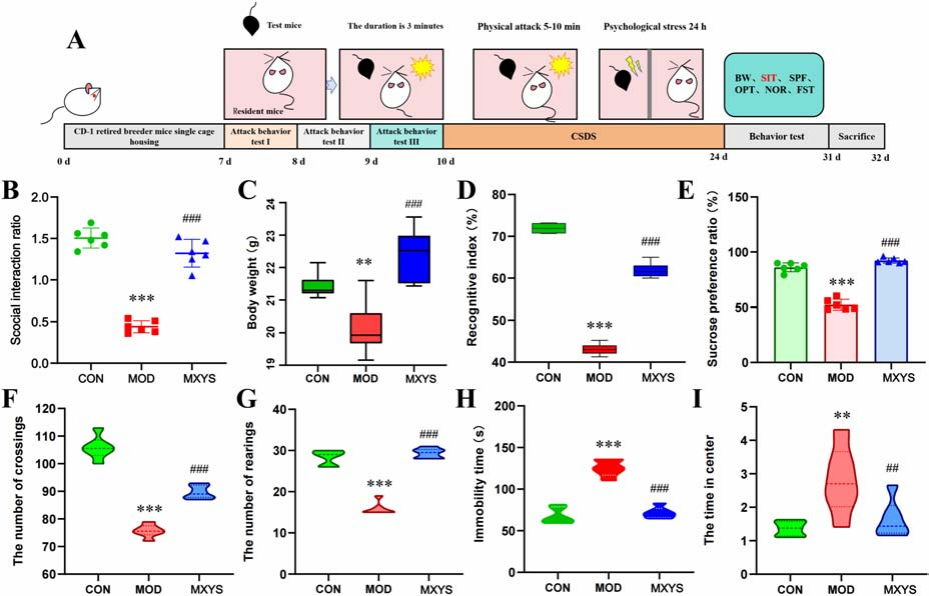
Procedure of animal experiments and efficacy evaluation of Modified Xiaoyaosan (MXYS). **(A)** Experimental procedures of chronic social defeat stress (CSDS) model. **(B)** Social interaction ratio. **(C)** Body weight. **(D)** Recognition index. **(E)** Sucrose preference ratio. **(F)** Number of crossings. **(G)** Number of rearings. **(H)** Immobility time. **(I)** Time in center. Values represent means ± SD. **P < 0.01, ***P < 0.001, compared with the CON group. ^##^*P* < 0.01, ^###^*P* < 0.001, compared with the MOD group (*n* = 6).

The CSDS procedure was performed as previously described (Zhao et al., 2025). Briefly, CD-1 aggressor mice were housed individually for 7 days to establish territorial awareness. Subsequently, C57BL/6N test mice were introduced into the CD-1 cage for 3 min per day. Aggression behavior was assessed based on two criteria: (a) more than five physical attacks, and (b) attack latency < 60 s. Only CD-1 aggressor mice that exhibited aggressive behavior toward test mice at least twice within a 3-day period were eligible for subsequent experiments. The experimental process is shown in Figure 1A.

Based on baseline performance in the open field test (OFT), sucrose preference test (SPT), and body weight, C57BL/6N test mice were randomly divided into three groups (*n* = 8): the CON group (without any stress), the MOD group (with CSDS stress), and the MXYS group (MXYS treatment with CSDS stress, 12.0 g herb/kg). The dosage of 12.0 g/kg was determined based on our previous dose-response experiment (Zhao et al., 2025), which confirmed that it was the optimal dose of MXYS to improve the social avoidance behavior in CSDS mice.

### 2.4 Behavioral assessments and sample collection

The behavioral tests were conducted in the following sequence: open field test (OFT), novel object recognition test (NOR), social interaction test (SIT), sucrose preference test (SPT), and forced swimming test (FST). All behavioral tests were separated by at least a 24-h interval to ensure sufficient recovery time for the animals. Before each test, mice were acclimated to the testing room for 30 min. Detailed protocols are provided in the Supplementary material.

After the final behavioral test, mice were anesthetized. Blood was collected from the retro-orbital plexus and centrifuged (3,500 rpm, 10 min, 4 °C) to obtain serum, and the hippocampus tissues were dissected on ice. The intestinal tract was carefully excised and segmented into the rectum, colon, ileum, jejunum, and duodenum from distal to proximal. Intestinal contents were gently removed to avoid mucosal damage, and almost 1 cm segment from each region was fixed in 4% paraformaldehyde. The remaining samples were snap-frozen in liquid nitrogen and stored at −80 °C for subsequent analysis.

### 2.5 Hematoxylin-eosin (HE) staining and periodic acid–Schiff (PAS) staining

Formalin-fixed intestinal tissues were embedded in paraffin and sectioned at 4 μm thickness. For morphological evaluation, sections were stained with hematoxylin and eosin (HE) using an automated stainer. To assess glycogen content, periodic acid–Schiff (PAS) staining was performed: sections were oxidized in 0.5% periodic acid for 10 min, incubated with Schiff’s reagent for 15 min, and counterstained with hematoxylin. Diastase-digested (PAS-D) controls were processed in parallel to confirm glycogen specificity.

### 2.6 16 S rRNA gene sequencing of intestinal microbiota

Intestinal contents were collected individually from each mouse. Microbiota analysis was performed by Shanghai Paison Biotechnology Co., Ltd. (Shanghai, China). Total bacterial genomic DNA was extracted, and the full-length 16S rRNA gene was amplified and sequenced using the PacBio Sequel platform. Species-level relative abundance profiles were generated for each sample, and the species composition was visualized using relative abundance bar plots. The 16S rRNA amplicon sequencing data were processed using QIIME 2 with the DADA2 plugin for denoising, dereplication, and chimera filtering, the parameters seen in Supplementary material. The α diversity level of microbial communities of each sample were assessed using the Chao1, Observed species, Shannon, and Simpson index. The β diversity among groups was analyzed by non-metric multidimensional scaling (NMDS) and hierarchical clustering analysis. The data presented in the study are deposited in the NCBI repository, accession number is PRJNA1393409.

### 2.7 Untargeted metabolomics of intestinal contents

#### 2.7.1 Sample preparation

Intestinal content samples (50 mg) were homogenized in 500 μL of acetonitrile: water (4:1, v/v) solution. After centrifugation at 13,000 rpm for 15 min at 4 °C, the supernatant was filtered through a 0.22 μm membrane. An aliquot of 10 μL from each sample was pooled to prepare quality control (QC) samples for both colonic and jejunal content analyses.

#### 2.7.2 LC-MS analysis

All samples were analyzed on a Thermo DIONEX UltiMate 3,000 high performance liquid chromatograph coupled with a Q-Exactive Hybrid Quadrupole-Orbitrap mass spectrometer (Thermo Fisher Scientific, Waltham, MA, United States). Chromatographic separation was performed on Waters Acquity UPLC HSS T3 column (2.1 mm × 100 mm, 1.7 μm) maintained at 40 °C. The mobile phase consisted of (A) 0.1% formic acid water and (B) 0.1% formic acid acetonitrile. The injection volume was 5 μL, and the flow rate was 0.2 mL/min. Mass spectrometry was conducted in both positive and negative HESI mode with key parameters set as follows: spray voltage, 3.5 kV (ESI+) and 2.5 kV (ESI−); capillary temperature, 320 °C; and a full scan range from 80 to 1,200 (m/z) at a resolution of 35,000. For detailed protocols, please refer to our previous study (Zhao et al., 2025). Metabolomics data processing was performed using Compound Discoverer 3.1 (Thermo Fisher Scientific), the parameters seen in Supplementary material. The untargeted metabolomics raw UPLC-MS/MS files have been deposited to iProX^1^ under accession number IPX0017187001.

### 2.8 Enzyme-linked immunosorbent assay (ELISA)

The level of interleukin-1β (IL-1β) and tumor necrosis factor-α (TNF-α) in serum, colon, and jejunum homogenates was measured using ELISA kits according to the manufacturer’s protocols. Similarly, the level of lipopolysaccharide (LPS), D-lactate (D-Lac), and diamine oxidase (DAO) in serum, as well as the level of carnosine and carnosine synthase 1 in colonic content, were determined using corresponding ELISA kits.

### 2.9 Western blot analysis

Intestinal tissues were performed on ice using RIPA buffer containing 1% (v/v) protease and phosphatase inhibitors. After homogenization, samples were incubated on ice for 30 min with vortexed for 1 min per 10 min, followed by centrifugation at 13,000 rpm for 15 min at 4 °C. Protein concentrations were determined using a BCA protein assay kit (Shark Biotech, China). Proteins (30 μg) were separated by SDS-PAGE and transferred onto PVDF membranes. Membranes were blocked with 5% non-fat milk in TBST for 2 h at room temperature, and then incubated overnight at 4 °C with primary antibodies: claudin-1 (1:1000), occludin (1:1000), and GAPDH (1:10000). After washing, membranes were incubated with HRP-conjugated secondary antibodies for 2 h at room temperature. Protein bands were visualized using enhanced chemiluminescence (ECL) reagent (Dalian Meilun Biotechnology, China) and quantified with ImageJ software (NIH, United States). GAPDH served as the loading control.

### 2.10 Molecular docking analysis

Molecular docking was performed to predict potential binding interactions between candidate metabolites and tight junction proteins. The three-dimensional structures of mouse claudin-1 (UniProt ID: O88551) and occludin (UniProt ID: Q9Z0T1) were retrieved from the AlphaFold Protein Structure Database. The predicted local distance difference test (pLDDT) scores were 84.62 for claudin-1 and 77.06 for occludin, indicating high confidence for most regions. The structures of aspartate and 1-linoleoyl-2-hydroxy-sn-glycero-3-PC were downloaded from PubChem and energy-minimized using MMFF94 force field in OpenBabel. Docking simulations were conducted using AutoDock Vina 1.2.0. Binding conformations with the lowest free energy were selected for visualization using PyMOL 2.5.

### 2.11 Statistical analysis

Statistical analysis was performed using GraphPad Prism 8.0 (GraphPad Software Inc., United States). Differences between two groups were assessed using Student’s *t*-test for normally distributed data; otherwise, the Mann-Whitney U test was applied. *P* < 0.05 was considered statistically significant. Differential metabolites were identified using orthogonal partial least squares discriminant analysis (OPLS-DA) with variable importance in projection (VIP) > 1.0. For univariate analysis, *P*-values were corrected for multiple testing using the Benjamini-Hochberg false discovery rate (FDR) method. Metabolites with FDR-adjusted q < 0.05 and VIP > 1.0 were considered statistically significant. Effect sizes were calculated for all comparisons. For two-group comparisons, Cohen’s d was computed (thresholds: small = 0.2, medium = 0.5, large = 0.8).

## 3 Results

### 3.1 MXYS ameliorates depressive-like and social avoidance behaviors in CSDS mice

Compared with the CON group, the MOD group exhibited significant reductions in social interaction ratio (Figure 1B), body weight (Figure 1C), recognition index (Figure 1D), sucrose preference ratio (Figure 1E), number of crossings (Figure 1F), and number of rearings (Figure 1G). Meanwhile, immobility time (Figure 1H) and the time in center (Figure 1I) were significantly increased. These behavioral alterations confirmed the successful induction of both depressive-like and social avoidance behaviors in CSDS mice. Administration of MXYS significantly reversed all these behavioral deficits, indicating its potent antidepressant and prosocial effects.

### 3.2 MXYS alleviates CSDS-induced intestinal histopathological damage

**FIGURE 2:**
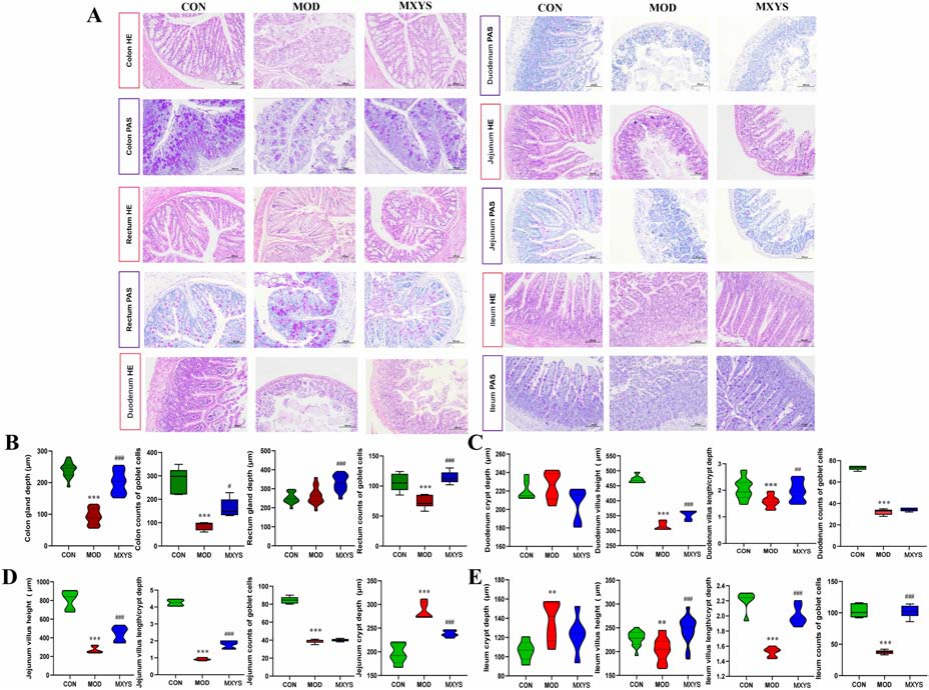
Modified Xiaoyaosan (MXYS) reduced histological damage of intestine in chronic social defeat stress (CSDS) mice. **(A)** The hematoxylin-eosin (HE) and periodic acid–Schiff (PAS) staining of the colon, rectum, duodenum, jejunum and ileum. **(B)** Colon gland depth and counts of goblet cells; Rectum gland depth and counts of goblet cells. **(C)** Duodennum villus height, crypt depth, villus height/crypt depth, and counts of goblet cells. **(D)** Jejunum villus height, crypt depth, villus height/crypt depth, and counts of goblet cells. **(E)** Duodennum villus height, crypt depth, villus height/crypt depth, and counts of goblet cells. Values represent means ± SD (*n* = 6). **P < 0.01, ***P < 0.001, compared with the CON group. ^#^*P* < 0.05, ^##^*P* < 0.01, ^###^*P* < 0.001, compared with the MOD group.

Intestinal morphological changes were evaluated by HE and PAS staining. Compared with the CON group, MOD group showed pronounced structural alterations in both the large and small intestines. In the large intestine, CSDS mice exhibited thinning of the muscle layer, disruption of the brush border, epithelial cell detachment, reduced goblet cell density, and shallowing of intestinal glands. In the small intestine, CSDS mice showed villus atrophy, crypt hyperplasia, decreased villus-to-crypt ratio, and lymphoid infiltration in the lamina propria. MXYS treatment markedly attenuated these pathological changes. Quantitative analysis using Image J further indicated that the colon and jejunum were particularly vulnerable to CSDS-induced damage. Our findings suggested that intestinal barrier dysfunction may contribute to depression pathogenesis and MXYS likely exerts its therapeutic effect through restoring intestinal integrity (Figure 2).

### 3.3 MXYS improves intestinal permeability and reduces inflammatory cytokine levels

**FIGURE 3:**
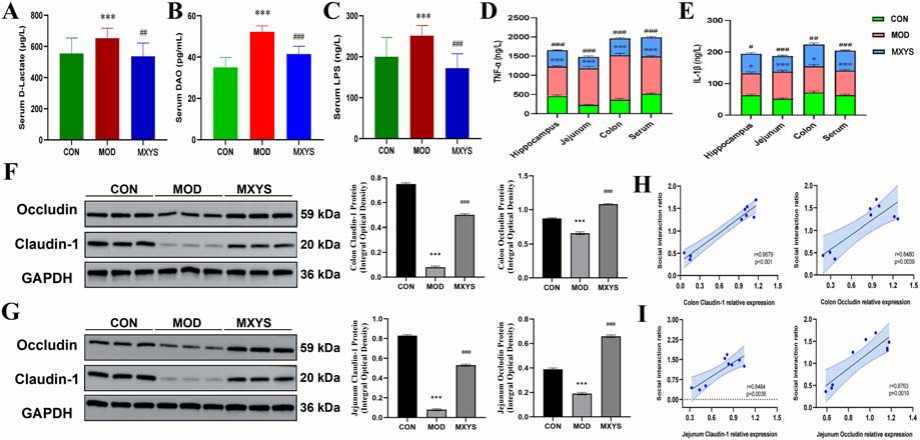
Effects of Modified Xiaoyaosan (MXYS) on permeability biomarkers, tight junction proteins and inflammatory factors in chronic social defeat stress (CSDS) mice. **(A–C)** Serum permeability related indicators. **(D)** The levels of TNF-α. **(E)** The levels of IL-1β. **(F,G)** The relative expression of claudin-1 and occludin in colon and jejunum. **(H,I)** Pearson correlation analysis of social avoidance behavior with intestinal tight junction proteins. Values represent means ± SD. *P < 0.05, ***P < 0.001, compared with the CON group. ^#^*P* < 0.05, ^##^*P* < 0.01, ^###^*P* < 0.001, compared with the MOD group (*n* = 4).

Given the link between intestinal permeability, systemic inflammation, and depression, we assessed serum permeability markers and inflammatory factors. ELISA results showed that serum levels of LPS, D-Lac, and DAO were significantly elevated in the MOD group compared with the CON group (Figures 3A–C and Supplementary Table 3). MXYS administration significantly reduced these markers. Furthermore, levels of the pro-inflammatory cytokines TNF-α and IL-1β were significantly increased in the hippocampus, jejunum, colon, and serum of MOD group (Figures 3D, E). Treatment with MXYS effectively reduced these cytokines.

To corroborate the functional improvement in barrier integrity, we examined the expression of tight junction proteins claudin-1 and occludin in the colon and jejunum by Western blot. As shown in Figures 3F, G and Supplementary Figure 2, protein levels of both claudin-1 and occludin were significantly higher in the MXYS group than in the MOD group, consistent with the observed reductions in serum permeability markers.

To compare the baseline expression of tight junction proteins between the colon and jejunum, we analyzed claudin-1 and occludin levels in CON group mice. As shown in Supplementary Figure 3, there were no significant differences in either claudin-1 or occludin protein expression between the colon and jejunum under basal conditions (*P* > 0.05). These findings support the segment-specific regulation of MXYS to restore barrier function according to the different pathological conditions of each intestinal segment.

### 3.4 Correlation analysis links intestinal barrier proteins to social behavior

We performed Pearson correlation analysis to explore associations between tight junction protein expression in intestinal tissues and social avoidance behavior (Figures 3H, I and Supplementary Table 4). In intestinal tissues, colon claudin-1 exhibited a strong positive correlation with social interaction ratio (| r| = 0.9679, *P* < 0.001, 95% CI [0.888, 0.985]), and colon occludin also showed a significant correlation (| r| = 0.8480, *P* < 0.01, 95% CI [0.699, 0.958]). Similarly, jejunal claudin-1 (| r| = 0.8484, *P* < 0.001, 95% CI [0.749, 0.977]) and occludin (| r| = 0.8763, *P* < 0.01, 95% CI [0.606, 0.918]) were positively correlated with social interaction. These findings suggested that higher expression of intestinal tight junction protein is associated with improved social behavior, which suggested that MXYS may mediate the improvement in intestinal permeability and social behavior in CSDS mice.

### 3.5 MXYS may modulates gut microbiota composition in the colon and jejunum of CSDS mice

Given the established role of gut microbiota in depression pathogenesis and our histopathological observations, we hypothesized that jejunal and colonic microbiota are involved in depression-related pathophysiology. We performed full-length 16S rRNA gene sequencing on intestinal contents to investigate microbial alterations.

**FIGURE 4:**
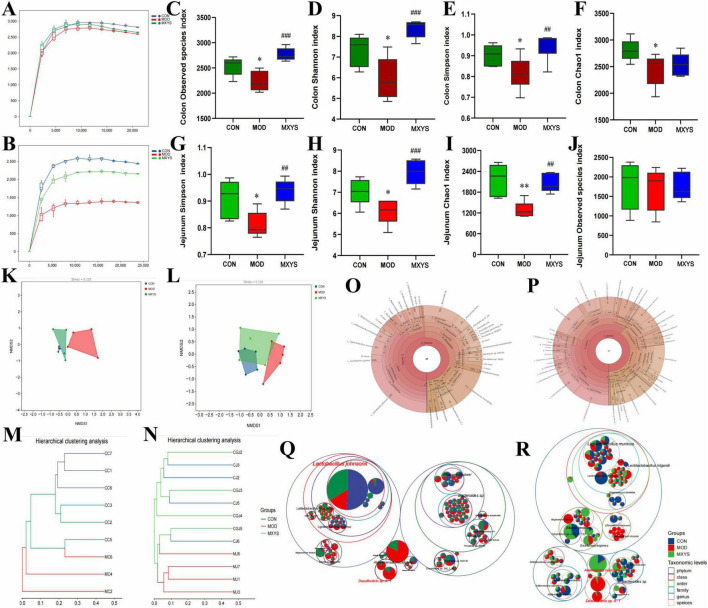
Gut microbiota diversity and composition analysis in colonic and jejunal contents. **(A)** Colonic content sparsity curve. **(B)** Jejunal content sparsity curve. **(C)** Colonic content observed species index. **(D)** Colonic content shannon index. **(E)** Colonic content simpson index. **(F)** Colonic content chao1 index. **(G)** Jejunal content simpson index. **(H)** Jejunal content shannon index. **(I)** Jejunal content chao1 index. **(J)** Jejunal content observed species index. **(K)** Colonic content non-metric multidimensional scaling analysis. **(L)** Jejunal content non-metric multidimensional scaling analysis. **(M)** Colonic content layer clustering analysis. **(N)** Jejunal content layer clustering analysis. **(O)** Colonic content species composition map. **(P)** Jejunal content species composition map. **(Q)** Colonic content bacteria species level classification dendrogram. **(R)** Jejunal content bacteria species level classification dendrogram. Values represent means ± SD. *P < 0.05, **P < 0.01, compared with the CON group. ^##^*P* < 0.01, ^###^*P* < 0.001, compared with the MOD group (*n* = 6).

Rarefaction curves plateaued with increasing sequencing depth, indicating adequate sampling coverage for both colon and jejunum samples (Figures 4A, B). Alpha diversity analysis revealed that CSDS mice significantly decreased the Shannon and Simpson indices compared with the CON group (Figures 4C–J), suggesting reduced microbial diversity. Beta diversity, assessed by NMDS (Figures 4K, L) and hierarchical clustering (Figures 4M, N), showed clear separation among the CON, MOD, and MXYS groups. Compared with the CON group, the microbial structure in CSDS mice was distinctly altered, whereas MXYS intervention restored the microbial homeostasis.

To assess whether the colon and jejunum harbor distinct microbial communities in our model, we performed NMDS analysis based on Bray-Curtis distance on the 16S rRNA sequencing data from colonic and jejunal contents within each treatment group (CON, CSDS, and MXYS). As shown in Supplementary Figure 4, colonic and jejunal microbiota clustered separately, indicating that these two intestinal segments harbor distinct ecological niches. The Bray-Curtis distance between colon and jejunum was significantly larger than within-segment distances (PERMANOVA, *P* < 0.001). In the CON group, colon and jejunum formed distinct clusters, indicating that compartmentalized microbiota distribution exists under normal physiological conditions. This separation remained significant in the CSDS group, confirming that stress exposure does not abolish inherent segment-specificity. Notably, the MXYS treatment maintained segmental divergence while shifting overall composition toward the CON group. These results demonstrate that the colon and jejunum constitute distinct microbial ecological niches in our CSDS mouse model, providing a basis for the segment-specific microbiota-metabolite networks.

Taxonomic composition analysis at the phylum level indicated that Bacillota (formerly Firmicutes) was the most abundant phylum in both colon and jejunum, while Pseudomonadota (formerly Proteobacteria) was the least prevalent. At the genus level, *Lactobacillus* dominated in the colon, whereas *Ligilactobacillus* was predominant in the jejunum (Figures 4O–R). Notably, the classification dendrogram revealed a significantly higher abundance of *Desulfovibrio* in both colonic and jejunal contents of the MOD group compared with the CON group. MXYS treatment suppressed these potentially pathogenic bacteria while enriching beneficial bacteria such as *Lactobacillus johnsonii* and *Akkermansia muciniphila*, indicating a restoration of microbial homeostasis.

**FIGURE 5:**
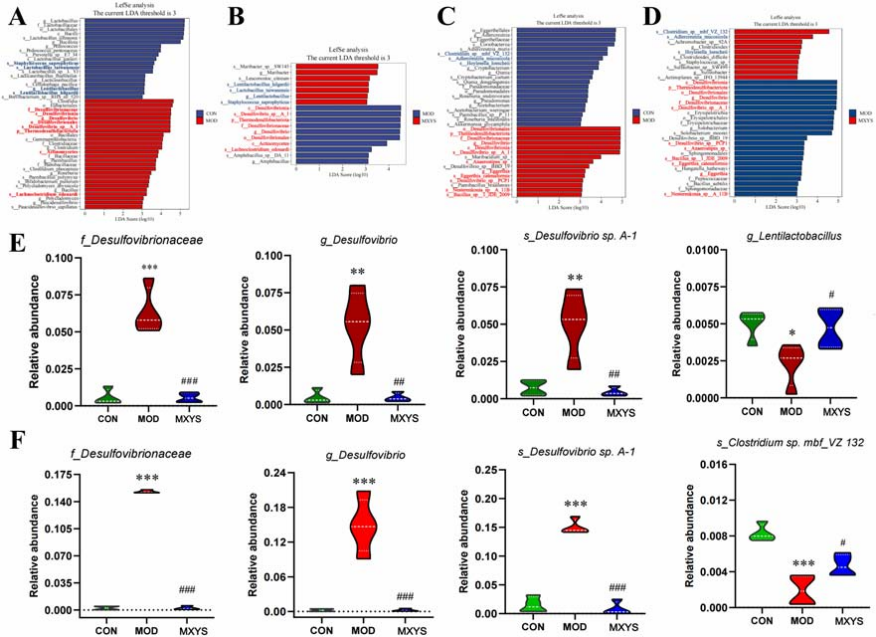
Differentially abundant microbial taxa identified in colonic and jejunal contents. **(A)** Species enriched in the colonic contents of the CON group compared with the MOD group. **(B)** Species enriched in the colonic contents of the Modified Xiaoyaosan (MXYS) group compared to the MOD group. **(C)** Species enriched in the jejunal contents of the CON group compared to the MOD group. **(D)** Species enriched in the jejunal contents of the MXYS group compared to the MOD group. **(E)** Key microbial marker species identified in colonic contents across groups. **(F)** Key microbial marker species identified in jejunal contents across groups. Values represent means ± SD. *P < 0.05, **P < 0.01, ***P < 0.001, compared with the CON group. ^#^*P* < 0.05, ^##^*P* < 0.01, ^###^*P* < 0.001, compared with the MOD group (*n* = 6).

Linear discriminant analysis effect size (LEfSe) analysis identified differentially abundant bacterial taxa among groups (Figures 5A–D). In the colon, compared with the MOD group, the CON and MXYS groups shared four bacterial taxa (blue labels): *Staphylococcus saprophyticus*, *Lactobacillus taiwanensis*, *Lentilactobacillus hilgardii*, and *Lentilactobacillus*. In the jejunum, three bacterial taxa were shared between the CON and MXYS groups: *Clostridium sp. mbf VZ-132*, *Adlercreutzia mucosicola*, *Hoylesella_loescheii*. Notably, six microbial taxa were consistently elevated in colonic and jejunal contents in the MOD group: *Thermodesulfobacteriota*, *Desulfovibrionia*, *Desulfovibrionales*, *Desulfovibrionaceae*, *Desulfovibrio*, and *Desulfovibrio sp. A-1*. These findings suggested that chronic stress promotes a dysbiotic state characterized by the expansion of pathobionts, which may compromise intestinal barrier function and increase permeability.

Abundance analysis further confirmed that the MOD group exhibited a significant increase in the relative abundance of *Desulfovibrionaceae*, *Desulfovibrio*, and *Desulfovibrio sp. A-1* in both colon and jejunum. Conversely, beneficial bacteria such as *Lentilactobacillus* in the colon and *Clostridium sp. mbf VZ-132* in the jejunum were significantly reduced (Figures 5E, F). MXYS intervention reversed these changes, reducing pathogenic bacteria and restoring beneficial genera.

PICRUSt2 functional prediction analysis suggested that the colonic and jejunal microbiota were primarily enriched in metabolic and genetic information processing pathways. Among these, four functional categories were notably altered: carbohydrate metabolism, cofactor and vitamin metabolism, amino acid metabolism, and lipid metabolism (Supplementary Figures 5A, B). However, as PICRUSt2 provides *in silico* predictions rather than direct functional measurements, future studies should employ shotgun metagenomic sequencing to directly assess the genetic potential of the gut microbiota, and metatranscriptomic analysis to determine which pathways are transcriptionally active.

### 3.6 MXYS may modulates colonic β-alanine and jejunal glycerophospholipid metabolism in CSDS mice

**FIGURE 6:**
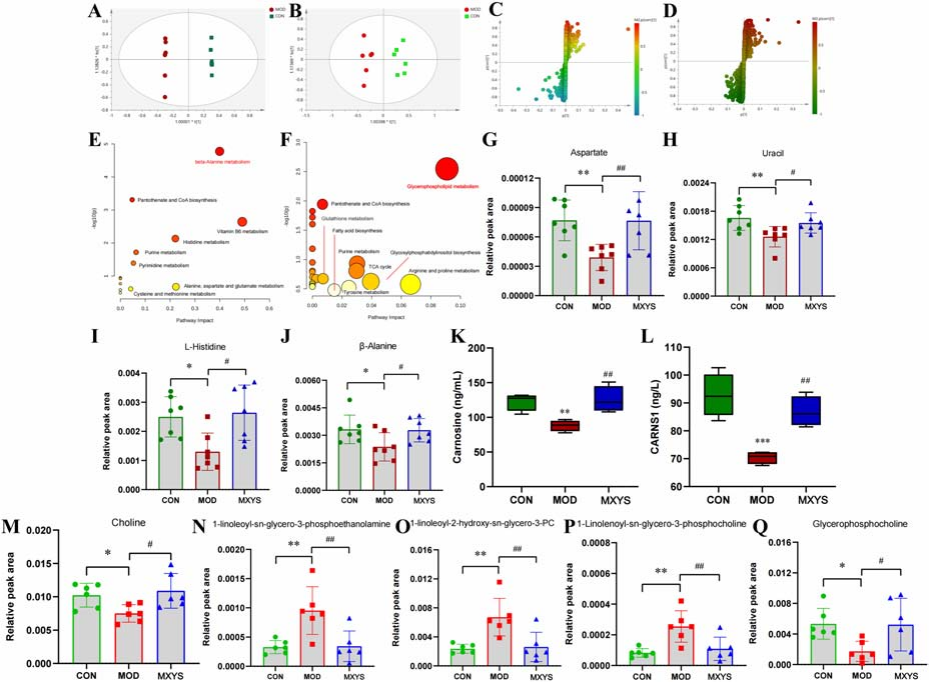
Metabolomic profiling and pathway analysis of colonic and jejunal contents. **(A,B)** Orthogonal partial least squares discriminant analysis (OPLS-DA) score plots. **(C,D)** S-plot of OPLS-DA model. **(E,F)** Metabolic pathway enrichment analysis of potential metabolic markers in colon and jejunum. **(G–J)** Potential metabolic markers related to β-alanine metabolism pathway in the colon (*n* = 7). **(K)** The level of carnosine (*n* = 6). **(L)** The level of carnosine (*n* = 6) synthetase 1. **(M–Q)** Potential metabolic markers related to glycerophospholipid metabolism pathway in the jejunum (*n* = 6). Values represent means ± SD. *P < 0.05, **P < 0.01, ***P < 0.001, compared with the CON group. ^#^*P* < 0.05, ^##^*P* < 0.01, compared with the MOD group.

To explore metabolic changes associated with CSDS-induced gut microbiota modulation, we performed untargeted metabolomic profiling of colonic and jejunal contents using UPLC-MS/MS in both positive and negative ion modes. Principal component analysis (PCA) revealed that all QC samples were clustered closely (Supplementary Figures 6A, B), confirming instrumental stability and high reproducibility of the analytical method. OPLS-DA revealed a significant separation between the CON and MOD groups (Figures 6A, B), indicating that chronic social defeat stress significantly altered the intestinal metabolite profile. Permutation tests (Supplementary Figures 6C, D) confirmed model validity without overfitting. Based on variable importance in projection (VIP > 1) and statistical significance (FDR-adjusted q < 0.05), we identified 21 potential biomarkers in colon (Supplementary Table 5) and 18 potential biomarkers in jejunum (Supplementary Table 6). Treatment with MXYS can significantly call back these biomarkers (Figures 6C, D).

Subsequently, KEGG pathway enrichment analysis revealed that the β-alanine metabolism pathway in the colon and the glycerophospholipid metabolism pathway in the jejunum were the most significantly altered pathways (Figures 6E, F). In the β-alanine metabolism pathway (Figures 6G–J), four key metabolite markers were identified: aspartate, uracil, β-alanine, and L-histidine. Meanwhile, carnosine and carnosine synthase 1, critical components of β-alanine metabolism, were also significantly reduced in the MOD group (Figures 6K, L). This may be related to the decrease of colonic tight junction protein expression and the increase of intestinal permeability. In the glycerophospholipid metabolism pathway (Figures 6M–Q), five metabolite markers were identified: choline, glycerophosphocholine, 1-linoleoyl-2-hydroxy-sn-glycero-3-PC, 1-linolenoyl-sn-glycero-3-phosphocholine, and 1-linoleoyl-sn-glycero-3-phosphoethanolamine.

### 3.7 Correlation analysis of the relationship between gut microbiota and its metabolites, intestinal permeability, and social avoidance behavior

**FIGURE 7:**
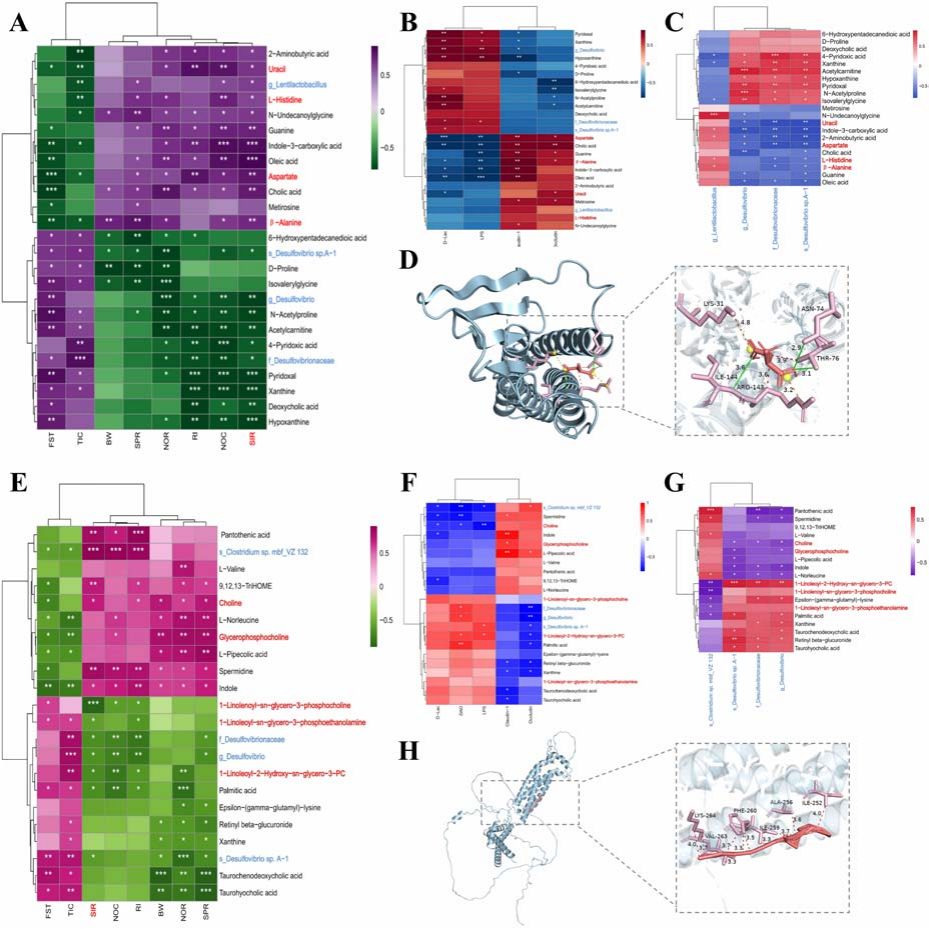
Integrative correlation analysis and molecular docking of microbiota, metabolites, intestinal barrier integrity, and social avoidance behavior in colon and jejunum. **(A)** Heatmap of the correlation between colonic marker species, potential metabolic markers, and social avoidance behavior. **(B)** Heatmap of the correlation between colonic marker species, potential metabolic markers, and intestinal permeability indices. **(C)** Correlation heatmap between colonic marker species and potential metabolic markers. **(D)** Molecular docking visualization of aspartate and claudin-1. **(E)** Heatmap of the correlation between jejunal marker species, potential metabolic markers, and social avoidance behavior. **(F)** Heatmap of the correlation between jejunal marker species, potential metabolic markers, and intestinal permeability indices. **(G)** Correlation heatmap between jejunal marker species and potential metabolic markers. **(H)** Molecular docking visualization of 1-linoleoyl-2-hydroxy-sn-glycero-3-PC and occludin. *P < 0.05, **P < 0.01, ***P < 0.001.

Spearman correlation analysis was performed to elucidate potential functional links between the gut microbiota, metabolites, intestinal permeability, and social avoidance behavior. The results revealed that aspartate was negatively correlated with social avoidance behavior (|r| = 0.79, *P* < 0.01), whereas the relative abundance of *Desulfovibrio* showed a strong positive correlation in colon (|r| = 0.78, *P* < 0.01). (Figure 7A). Meanwhile, *Desulfovibrio* abundance was positively correlated with the markers of intestinal permeability (LPS, |r| = 0.83, *P* < 0.01; D-Lac |r| = 0.87, *P* < 0.01) (Figure 7B), and negatively correlated with the tight junction protein claudin-1 (|r| = 0.73, *P* < 0.05). Additionally, aspartate showed a significant negative correlation with LPS (|r| = 0.83, *P* < 0.01) and D-Lac (|r| = 0.93, *P* < 0.001), and a significant positive correlation with claudin-1 (|r| = 0.83, *P* < 0.01) and occludin (|r| = 0.82, *P* < 0.05).

We further analyzed associations between potential metabolic markers and microbial taxa (Figure 7C). Aspartate was inversely correlated with *Desulfovibrio* abundance. Together with Pearson correlations linking tight junction proteins to social avoidance behavior, these results identified aspartate maybe a key metabolic marker and *Desulfovibrio* maybe a pivotal microbial taxon jointly associated with intestinal permeability and social avoidance.

Similarly, spearman correlation analysis revealed that the metabolite marker 1-linoleoyl-2-hydroxy-sn-glycero-3-PC was negatively correlated with social avoidance behavior (|r| = 0.70, *P* < 0.05), while *Clostridium sp. mbf VZ-132* exhibited a strong positive correlation with social avoidance behavior (|r| = 0.90, *P* < 0.001) (Figure 7E). Meanwhile, the metabolite marker was positively correlated with the markers of intestinal permeability (LPS, |r| = 0.78, *P* < 0.05; DAO, |r| = 0.77, *P* < 0.05), and a negative correlation with occludin (|r| = 0.78, *P* < 0.05). *Clostridium sp. mbf VZ-132* exhibited a negative correlation with the markers of intestinal permeability (LPS, |r| = 0.82, *P* < 0.05; D-Lac, |r| = 0.77, *P* < 0.05), while positively correlated with the tight junction protein occludin (|r| = 0.77, *P* < 0.05) (Figure 7F). The metabolite marker was also negatively associated with the abundance of *Clostridium sp. mbf VZ-132* (Figure 7G).

Molecular docking was performed to explore potential direct interactions between candidate metabolites and tight junction proteins. Redocking validation yielded RMSD values of 1.42 Å for the aspartate-docking protocol and 1.68 Å for the LPC-docking protocol, indicating acceptable reproducibility. Aspartate showed a predicted binding affinity of −4.5 kcal/mol to claudin-1, and key interacting residues included THR-76, LYS-31, ILE-144, ARG-143, and ASN-74 (Figure 7D). While 1-linoleoyl-2-hydroxy-sn-glycero-3-PC showed a higher binding affinity of −5.2 kcal/mol to occludin, and key binding residues for this interaction included LYS-264, VAL-263, PHE-260, ILE-259, ILE-252, and ALA-256 (Figure 7H). These values suggest weak-to-moderate binding strengths, consistent with transient, regulatory interactions between small metabolites and membrane proteins rather than high-affinity stable complexes. Collectively, these results imply that the *Desulfovibrio*_aspartate_claudin-1 axis and *Clostridium* sp. mbf VZ-132_1-linoleoyl-2-hydroxy-sn-glycero-3-PC_occludin axis may be involved in MXYS improving intestinal barrier function and social avoidance behavior in CSDS mice.

## 4 Discussion

### 4.1 MXYS may ameliorates social avoidance by restoring gut barrier integrity and reducing neuroinflammation

**FIGURE 8:**
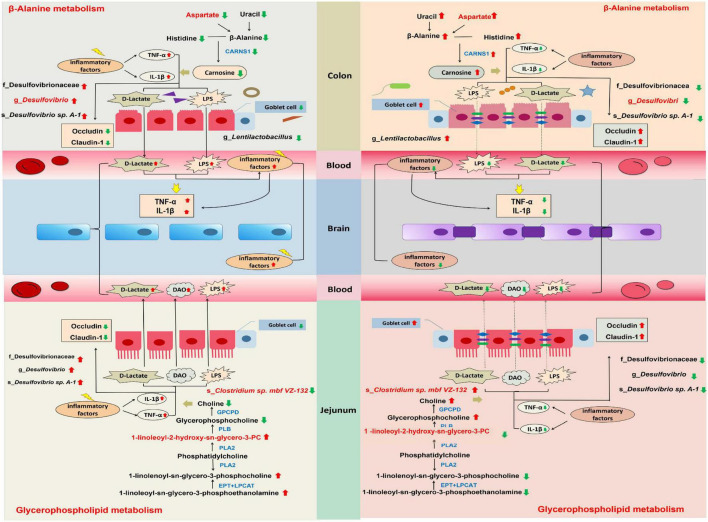
Graphic summary of the antidepressant effects and mechanisms of Modified Xiaoyaosan (MXYS).

The gut-brain axis has recognized as a pivotal framework for elucidating the pathophysiology of major depressive disorder (MDD), particularly its core behavioral symptom of social avoidance (Creed, 2022; He et al., 2025; Wu et al., 2026). Our study provided robust experimental evidence that MXYS may alleviated social avoidance behavior in CSDS mice by modulating the gut microbiota and their metabolites (Figure 8). These alterations collectively enhanced intestinal barrier function and reduced permeability. Notably, this targeted regulation of gut microbiota and barrier integrity may constitute a key pharmacological foundation for alleviating social avoidance behaviors in depression.

Our investigation pinpointed the colon and jejunum as primary sites of CSDS-induced pathological damage, characterized by villus atrophy, crypt hyperplasia, goblet cell depletion, and inflammatory infiltration. These structural impairments translated into a functional breakdown of the intestinal barrier, as evidenced by elevated serum levels of LPS, D-Lac, and DAO, established markers of intestinal hyperpermeability and bacterial translocation (Raman et al., 2023; Xie et al., 2023). MXYS administration effectively restored mucosal architecture and normalized these permeability markers. This improvement in gut barrier function was paralleled by a significant upregulation of the key tight junction proteins claudin-1 and occludin in intestine. This coordinated restoration suggested a systemic barrier-stabilizing effect of MXYS, potentially stemming from reduced systemic inflammation, as reflected by decreased levels of TNF-α and IL-1β in both gut and brain. The strong positive correlation between the expression of intestinal claudin-1/occludin and social interaction behavior underscores a potential link between gut barrier integrity and behavioral outcome.

The convergence of these gut-centric mechanisms on the brain is highlighted by improved hippocampal barrier proteins and reduced neuroinflammation. Increased intestinal permeability permits translocation of microbial products like LPS into circulation, triggering a peripheral immune response that can compromise the blood-brain barrier and activate microglia, leading to neuroinflammation (Barichello et al., 2022). The observed reductions in hippocampal TNF-α and IL-1β indicated MXYS mitigates this neuroinflammatory cascade. Therefore, MXYS interrupts a vicious cycle wherein stress-induced gut dysbiosis and barrier failure promote systemic and neuroinflammation, exacerbating behavioral deficits.

### 4.2 Segment-specific remodeling of gut microbiota and metabolites by MXYS

Chronic stress-induced dysbiosis was characterized by a marked expansion of *Desulfovibrionaceae*, particularly *Desulfovibrio*, in both the colon and jejunum. Studies have shown that *Desulfovibrio* increases intestinal permeability in high-fat-fed mice by reducing the expression of tight junction proteins and the thickness of the mucus layer (Li et al., 2022). Similarly, our study confirmed that enrichment of *Desulfovibrionaceae* in the colon and jejunum impaired the expression of tight junction proteins and elevated intestinal inflammatory factors. The positive correlation between *Desulfovibrio* abundance and serum permeability markers, coupled with its negative association with tight junction protein expression, strongly implicated this bacterial group in stress-related barrier disruption. MXYS treatment effectively suppressed this pathogenic expansion while differentially enriching beneficial commensals. Notably, the enrichment of *Clostridium sp. mbf VZ-132* aligns with the known role of related butyrate-producing clostridia in reinforcing intestinal barrier integrity and exerting anti-inflammatory, antidepressant effects (Recharla et al., 2023; Zhang et al., 2023), suggesting a conserved beneficial function within this phylogenetic group. Meanwhile, our findings demonstrated that the structure and dynamics of the gut microbiota are not homogeneous, but exhibit considerable segment-specificity. Due to the distinct physiological environments in segments such as the colon and jejunum, which result in fundamental differences in microbial composition and variation patterns.

Recent studies emphasized the crucial influence of gut microbial metabolites on social avoidance behavior (Li et al., 2022). Our metabolomic analysis identified the β-alanine metabolism pathway was the most significantly altered in the colon of CSDS mice, and aspartate, a direct precursor for β-alanine metabolism, was markedly reduced. Correlation network showed *Desulfovibrio* abundance negatively correlated with aspartate, whereas aspartate positively correlated with claudin-1/occludin expression and negatively correlated with permeability markers and social avoidance. However, these computational predictions require experimental validation using techniques such as surface plasmon resonance or mutagenesis studies. We proposed that MXYS inhibits *Desulfovibrio* expansion, thereby restoring aspartate availability, which could potentially enhance tight junction integrity either through direct protein interaction (as suggested by our molecular docking analysis) or indirectly via the synthesis of protective metabolites such as carnosine. The direct interaction hypothesis remains speculative and requires biochemical validation.

Recent research has indicated that aspartate can improve intestinal health and mitigate oxidative stress by modulating gut microbiota (Hernández-Cacho et al., 2025). These findings provide important correlational evidence linking gut dysbiosis to depressive disorders. However, as a cross-sectional study, it could not determine whether these disturbances are causes of depression, nor whether they can be reversed by intervention. Comparison with this human cross-sectional study, our findings complement and extend recent human multi-omics studies in depression. In our study, we demonstrate that MXYS treatment directionally restores CSDS-induced reductions in microbial diversity and differentially regulates specific taxa in a segment-specific manner. Our segment-specific analysis revealed that the colon and jejunum harbor distinct microbial communities and exhibit different metabolic responses to stress and intervention, which can not accessible in human studies.

In the jejunum, metabolomic profiling also revealed significant alterations in glycerophospholipid metabolism, with the 1-linoleoyl-2-hydroxy-sn-glycero-3-PC being markedly elevated in CSDS mice. This specific lysophosphatidylcholine (LPC) displayed pro-inflammatory and barrier-disrupting properties (Tan et al., 2020; Jin S. et al., 2025), showing a positive correlation with intestinal permeability markers and social avoidance, and a negative correlation with the tight junction protein occludin. Conversely, it negatively correlated with the abundance of *Clostridium sp. mbf VZ-132*. Molecular docking predicted a weak-to-moderate affinity interaction between LPC and occludin, suggesting a potential interference with tight junction integrity. Thus, MXYS appeared to enhance jejunal barrier function by promoting *Clostridium sp. mbf VZ-132*, which may facilitate the reduction of LPC and alleviate the inhibitory effect on occludin, thereby improving social behavior.

### 4.3 Differential regulation of tight junction proteins and segment-specific networks

As a principal structural component of tight junctions, claudin-1 is critical for establishing the intercellular permeability barrier and governing paracellular transport (Li et al., 2016; Shokravi et al., 2025). Studies in lung cancer cells associate claudin-1 overexpression with enhanced invasiveness, while knockdown of claudin-1 resulted in increased invasion and metastasis (Jiang et al., 2017). Intriguingly, we observed a positive correlation between claudin-1 expression and aspartate levels, suggesting aspartate may promote claudin-1 expression under depressive conditions to reduce colonic permeability and ameliorate social avoidance behavior. Occludin, another key tight junction protein, interacts with claudins to form a physical barrier restricting paracellular transport (Kage et al., 2019). Impaired occludin function compromises tight junction integrity, leading to increased intestinal permeability (Kuo et al., 2019). LPC, as a phospholipid metabolic intermediate, reflects cellular metabolic status (Balta et al., 2022). Elevated LPC under metabolic disturbance may inhibit occludin synthesis by modulating intracellular signaling (Tang et al., 2021). Our analysis revealed a positive correlation between occludin expression and LPC levels, suggesting MXYS intervention may modulate LPC levels, reducing jejunal permeability and alleviating social avoidance behavior.

Of note, our study revealed a marked segment-specific microbiota–metabolite association network between the colon and jejunum. The colon was primarily involved in β-alanine metabolism, whereas the jejunum was mainly engaged in glycerophospholipid metabolism. This segmental difference is not coincidental, but is closely related to the distinct physiological microenvironments, microbial colonization preferences, metabolite absorption characteristics, and barrier function heterogeneity along the intestinal tract. Firstly, it has been demonstrated that the microbiota exhibits compartmentalized distribution along the gastrointestinal tract, with microbes from different segments tending to colonize their homologous sites (Li et al., 2020). This underpins the fundamental differences in microbial composition and function between the colon and jejunum. Secondly, the capacity for metabolite absorption and transport differs across intestinal segments. It has been shown that the β-alanine treatment can increase intestinal epithelial occludin expression and improve barrier function (Park et al., 2024). In the colon, β-alanine and its precursor aspartate are primarily derived from microbial metabolism and can act locally on the tight junction protein claudin-1 (Salvo Romero et al., 2015). In contrast, the jejunum is the central site for lipid digestion and absorption, where glycerophospholipids are hydrolyzed into lysophosphatidylcholine (LPC) in the intestinal lumen and then absorbed by enterocytes (Nakano et al., 2009). Therefore, abnormal changes in metabolic intermediates are more readily detected in this segment. Thirdly, the composition and regulatory mechanisms of tight junction proteins themselves are heterogeneous between the colon and jejunum. Studies have shown that claudin family proteins exhibit a gradient expression pattern along the longitudinal axis of the intestine, whereas occludin expression is relatively stable (Holmes et al., 2006). Although our baseline data showed no significant differences in the protein expression of claudin-1 or occludin between the colon and jejunum in the CON group, the composition and regulatory mechanisms of tight junction proteins can still exhibit segment-specific heterogeneity under pathological conditions. This implies that the two intestinal segments may differ in their susceptibility to stress-induced injury and their capacity for repair. Such differential responsiveness provides a rationale for MXYS to exert its barrier-restoring effects through distinct microbiota–metabolite pathways in the colon versus the jejunum.

Clinical studies have also demonstrated significant disturbances in both glycerophospholipid metabolism and alanine/aspartate metabolism pathways in patients with major depressive disorder (Zu et al., 2024). While these human studies have established important associations between gut microbiota, glycerophospholipid/alanine-aspartate metabolism, and MDD, they cannot determine correlation relation or test the effects of therapeutic interventions. The present study aims to investigate whether MXYS can directionally restore the microbiota and metabolic disturbances observed in depression, and whether such restoration is linked to the amelioration of social avoidance behavior. Unlike human studies, our experimental design allows us to test the correlation of microbiota-metabolite-barrier axes based on CSDS mice and to reveal the segment-specific effects along the intestinal tract that are not accessible in human studies. In summary, this segment-specific remodeling of the microbiota and metabolites highlights the finely tuned regional division of labor established during host–microbe coevolution. It also provides a novel theoretical basis for understanding how traditional Chinese medicine formulas exert multi-target, multi-segment actions to integratively regulate the gut–brain axis.

### 4.4 Limitations and future perspectives

While our integrative analysis revealed significant correlations between gut microbial taxa and specific metabolites, it is impossible to definitively distinguish whether the different metabolites identified are derived from the gut microbiota, the host, or both. Stable-isotope tracing experiments or germ-free mouse validation would be required to establish causality and source attribution in future investigations. In addition, while our previous studies have demonstrated comparable efficacy between MXYS and the positive drug Paroxetine (Zhao et al., 2025; Wu et al., 2026), we acknowledge that the lack of a concurrent positive control group in the present study is a major limitation affecting efficacy benchmarking. Therefore, we state our plan to include a positive control to strengthen the evidence in future investigations.

While our study identified significant correlations among gut microbiota, metabolites, and intestinal barrier proteins, further experimental validation is required. Future studies should employ fecal microbiota transplantation (FMT) to test whether transfer microbiota recapitulates the behavioral and barrier phenotypes, a detailed FMT experimental design is shown in the Supplementary Figure 7. If the MXYS-modified microbiota is causally involved in alleviating social avoidance, FMT-MXYS recipients should exhibit improved social interaction behavior, reduced intestinal permeability markers, and upregulated tight junction protein expression compared to FMT-CSDS recipients. Conversely, FMT from vehicle-treated CSDS donors should not confer these beneficial effects. Additionally, direct metabolite intervention experiments are essential to establish causality for the proposed metabolite-protein-behavior links. Direct administration of aspartate or LPC would test whether this metabolite alone is sufficient to rescue social avoidance behavior and restore tight junction protein expression. The detailed experimental design is shown in the Supplementary material.

While docking analysis can suggest potential binding modes and affinities, it does not constitute evidence of *in vivo* protein-metabolite interactions. It is worth noting that the predicted binding affinities of aspartate for claudin-1 (−4.5 kcal/mol) and LPC for occludin (−5.2 kcal/mol) are in the weak-to-moderate range. This is physiologically plausible, as endogenous metabolites typically modulate protein function through transient, low-affinity interactions rather than permanent binding. This moderate affinity is in line with the characteristics of the interaction between signal molecules and membrane proteins, which may allow dynamic regulation of tight junction assembly and disassembly in response to metabolic signals. Future surface plasmon resonance or isothermal titration calorimetry experiments are warranted to experimentally validate these interactions and determine accurate binding constants.

## 5 Conclusion

Our findings suggested that MXYS is associated with segment-specific remodeling of gut microbiota and metabolites, which may contribute to the amelioration of social avoidance behavior through potential effects on intestinal barrier integrity. Specifically, in the colon, MXYS may reduced the abundance of *Desulfovibrio*, modulated aspartate biosynthesis in the β-alanine metabolism pathway, and upregulated claudin-1 expression. In the jejunum, MXYS promoted *Clostridium sp. mbf VZ-132*, downregulated 1-linoleoyl-2-hydroxy-sn-glycero-3-PC biosynthesis in the glycerophospholipid metabolism pathway, and enhanced occludin expression. Our research systematically investigated the antidepressant pharmacological effects and potential mechanisms of MXYS. These findings provide a novel theoretical framework and a targeted MGBA-based strategy for the treatment of psychiatric disorders.

## Data Availability

The 16S rRNA data presented in the study are deposited in the NCBI repository, accession number is PRJNA1393409. The untargeted metabolomics raw UPLC-MS/MS files are deposited to iProX (https://iprox.cn/) under accession number IPX0017187001.

## Footnotes

1
https://iprox.cn/
