# Supplementary material for: Intestinal microbial remodeling and metabolite regulation may be related to the improvement of social avoidance in depressed mice by Modified Xiaoyaosan
Source: Front Microbiol. 2026 Jul 27;17:1774975. doi: 10.3389/fmicb.2026.1774975 (PMC13454121; doi:10.3389/fmicb.2026.1774975)
Supplement: Supplementary file 1 [file Supplementary_file_1.docx]

**Intestinal microbial remodeling and metabolite regulation may be related to the improvement of social avoidance in depressed mice by Modified Xiaoyaosan**

**Brief description of the supporting information**

**Methods and materials**

1 Behavioral tests

2 Fecal bacteria transplantation experiment design

3 Metabolite intervention experiments

4 Parameters for 16S rRNA data processing

5 Parameters for untargeted metabolomics processing

**Figure**

Figure S1 Chromatogram of reference substance (A) and MXYS (B).

Figure S2 The uncropped membranes for all target proteins.

Figure S3 The baseline comparative for claudin-1 and occludin expression.

Figure S4 NMDS analysis of colonic and jejunal contents in different experimental groups.

Figure S5 Predicted functional profiling of bacterial communities in colonic and jejunal.

Figure S6 Quality control and multivariate statistical analysis.

Figure S7 The design of fecal bacteria transplantation experiment.

**Table**

Table S1 Content of Saikosaponin (a, b2, b1, d) in three batches of MXYS.

Table S2 Content of paeoniflorin in three batches of MXYS.

Table S3 Effects of behavioral and biochemical comparisons.

Table S4 Bootstrap validation of key correlations.

Table S5 Metabolic biomarkers in the colon content of mice.

Table S6 Metabolic biomarkers in the jejunum content of mice.

**Methods and materials**

**1 Behavioral tests**

All tested behaviors were observed in quiet environments under dim lighting.

**1.1 Body weight (BW)**

During the CSDS induction period, the body weights of mice were recorded at regular intervals. All rats in each group were weighed at baseline (0 weeks) and after 2 weeks of CSDS exposure. These measurements were used to assess the success of CSDS induction and its effects on body weight after administration.

**1.2 Social interaction test (SIT)**

The SIT was used to evaluate the social avoidance behavior of test mice exposed to CSDS. The experimental setup consisted of a standardized social-test chamber (50 cm × 50 cm × 25 cm), and the test procedure was divided into two stages: 1) The first stage (no social stimulation period), the test mice were placed individually in the chamber, with an empty transparent acrylic cage (10 cm diameter, 10 cm height) positioned on one side. Behavior was continuously recorded for 150 s. This phase was used to assess the baseline activity level of mice in the absence of social stimulation; 2) The second stage (social stimulation period), CD-1 retired breeder mice were placed in the acrylic cage, and the test mice were reintroduced into the chamber for 150 s . During the experiment, the time of test mice in the social interaction area (10 cm × 15 cm) was recorded. The key behavioral metric was the social interaction ratio (SIR), calculated as follows: SIR = (time spent in social interaction in stage 2) / (time spent in social interaction in stage 1). Based on established criteria, an SIR < 1 was defined as the threshold for social avoidance behavior, indicating that the test mice exhibited a significant social avoidance response in the presence of social stimulation. All behavioral tests were conducted within 24 hours after CSDS exposure to ensure the temporal consistency of stress effects.

**1.3 Forced swimming test (FST)**

The FST was used to assess behavioral desperation in test mice exposed to CSDS. A transparent cylindrical bucket (diameter: 10 cm, height: 30 cm) was filled with warm water at 25 ± 1°C, maintaining a depth of 15-20 cm to prevent the mice's tails from touching the bottom. The experiment consisted of two phases: acclimatization (2 min) and testing (4 min). First, the test mice were placed in the bucket and allowed to swim freely for 2 min to acclimate. Then, they were removed and briefly dried. After a 30-minute interval, the test was repeated, and immobility time (defined as floating without active struggling) was recorded within 4 min. Immediately after the test, the test mice were dried with a towel and returned to their cages. The water temperature was kept constant, and the water was replaced between groups to minimize esidual odor effects.

**1.4 Sucrose preference test (SPT)**

The SPT was used to assess anhedonia in test mice exposed to CSDS. During the training period, the test mice were housed individually and provided with two identical water bottles (1% sucrose solution on the left and pure water on the right). To control for spatial bias, the positions of the bottles were switched after 12 hours, followed by a 24-hour sucrose preference training phase. Next, a 12-hour deprivation period (water and food) was implemented to increase motivation to drink during testing. In the test phase, both the sucrose solution and pure water were available, and their consumption was measured separately after 12 hours. The sucrose preference ratio (the consumption of sucrose solution (g)/(the consumption of sucrose solution [g] + the consumption of pure water [g]) × 100%) served as the primary evaluation metric.

**1.5 Open field test (OFT)**

The OFT was used to assess the activity and exploratory ability in test mice exposed to CSDS. The experimental procedure consisted of a 1-minute acclimatization phase followed by a 4-minute testing phase. During the test, mice were placed in the center of a black polyethylene open-field box (50 cm × 50 cm × 40 cm) and allowed to move freely. An observer recorded the following behavioral parameters: 1) number of crossed grids (counted when a front paw entered an adjacent grid), reflecting horizontal locomotion; 2) number of rearings (defined as forelimbs leaving the ground or touching the box walls), indicating exploratory behavior.

**1.6 Novel object recognition (NOR)**

The NOR was used to assess the recognition and memory function of novel objects in test mice exposed to CSDS. The experiment consisted of two stages: a free exploration phase and a testing phase. In the free exploration phase, the mice were placed in a black polyethylene open-field box (50 cm × 50 cm × 40 cm) for 10 minutes of unrestricted exploration before being returned to their home cage. After a one-hour interval, the mice proceeded to the testing phase, where they were reintroduced to the open-field box, now containing two novel objects, and allowed to explore for 5 minutes. The time spent investigating each object was recorded.

**2 Fecal bacteria transplantation experiment design**

**2.1 Preparation of fecal bacteria suspension and sterile suspension**

The CSDS model was replicated, and the fecal samples of donor mice normal control group (CON) and CSDS group (MOD) and MXYS group (MXYS) were collected. According to the ratio of the weight of the fecal sample to the volume of the normal saline solution of 1:10 (w/v), the mixture was stirred evenly and vortexed for 5 min, and filtered with 200 mesh sterile gauze to obtain the coarse fecal suspension. The crude fecal bacteria suspension was centrifuged at a speed of 600 g for 5 min, and the supernatant was taken as the fecal bacteria suspension in the FMT experiment. The fecal suspension of CSDS mice treated with MXYS was sterilized in a high-pressure steam sterilizer at 121℃ for 20 min to obtain a sterile suspension of CSDS mice treated with MXYS.

**2.2 Grouping and process of fecal microbiota transplantation experiment**

After 7 days of adaptive feeding, C57BL/6N mice were interfered with mixed antibiotics. Refer to the previous method of our research group. The mice after 14 days of antibiotic intervention were randomly divided into 4 groups, 8 mice in each group received FMT. Details of each group are as follows :

(i) Blank fecal microbiota transplantation group (FCON): the fecal microbiota suspension of normal control group (CON) mice was received, containing normal intestinal flora;

(ii) CSDS fecal microbiota transplantation group (FCSDS): the fecal microbiota suspension of CSDS group (MOD) was received, containing the characteristic intestinal flora caused by CSDS stress;

(iii) MXYS fecal bacteria transplantation group (FMXYS): the fecal microbiota suspension of MXYS group (MXYS) was received, containing the characteristic intestinal flora after the intervention of MXYS;

(iiii) MXYS sterilization transplantation group (SFMXYS): the sterile fecal suspension of MXYS group (MXYS) was received, not containing the characteristics of intestinal flora after the intervention of MXYS.

Each mouse was given a corresponding fecal bacterial suspension or sterile suspension by gavage at a volume of 10 μL/g. The fecal microbiota transplantation process lasted for 3 weeks, 3 times a week. The detail experimental design is shown in Fig.S5.

**3 Metabolite intervention experiments**

Animal groups (n=8 per group): (1) CON + vehicle, (2) CSDS + vehicle, (3) CSDS + MXYS (positive control), (4) CSDS + aspartate, (5) CON + LPS. Exogenous aspartate and LPS via oral gavage for three weeks. The primary outcome measures include social interaction behavior, tight juntion protein expression, and serum levels of intestinal permeability markers (LPS, D-Lac, and DAO).

**4 Parameters for 16S rRNA data processing**

Microbiome bioinformatics were performed with QIIME 2 (2019.4) with slight modification according to the official tutorials (https://docs.qiime2.org/2019.4/tutorials/). Briefly, raw sequence data were demultiplexed using the demux plugin following by primers cutting with cutadapt plugin. Sequences were then quality filtered, denoised, merged and chimera removed using the DADA2 plugin. Non-singleton amplicon sequence variants (ASVs) were aligned with mafft and used to construct a phylogeny with fasttree2. Taxonomy was assigned to ASVs using the classify-sklearn naïve Bayes taxonomy classifier in feature-classifier plugin against the Greengenes 13_8 99% OTUs reference sequences.

**5 Parameters for untargeted metabolomics processing**

Metabolomics data processing was performed using Compound Discoverer 3.1 (Thermo Fisher Scientific). The workflow parameters were set as follows: precursor mass tolerance with 5 ppm; retention time alignment using adaptive curve model with 5.0 ppm mass tolerance and maximum shift of 0.5 min; compound detection with 5.0 ppm mass tolerance, 30% intensity tolerance, signal-to-noise ratio threshold of 1.5, and minimum peak intensity of 1,000. Compound grouping was performed with 5.0 ppm mass tolerance and 0.5 min retention time tolerance, with [M+H]^+^ and [M−H]^-^ as preferred ions. For spectral matching, mzCloud was searched using cosine similarity with a match factor threshold of 30; mzVault was searched with precursor mass tolerance of 5.0 ppm and FT fragment mass tolerance of 5 ppm. Final compound annotations were assigned using mzCloud, mzVault, and mass lists as data sources, with a mass tolerance of 5.0 ppm.

**Figure S1 Chromatogram of reference substance (A) and MXYS (B).** (1-Albiflorin, 2-Paeoniflorin, 3-Liquiritin, 4-Ferulic acid, 5-Monoammonium Glycyrrhizinate, 6-Saikosaponin A, 7-Saikosaponin B2, 8-Atractylenolide III, 9-Imperatorin, 10-Ligustilide, 11-Atractylenolide I, 12-2-Atractylenolide)

**Figure S2 The uncropped membranes for all target proteins.** (A) The protein expression of occludin in colon. (B) The protein expression of claudin-1 in colon. (C) The protein expression of occludin in jejunum. (D) The protein expression of claudin-1 in jejunum.

**Figure S3 The baseline comparative for claudin-1 and occludin expression.** (A) The protein expression of occludin in jejunum and colon of CON mice. (B) The protein expression of claudin-1 in jejunum and colon of CON mice. (C-D) The protein expression level of occludin and claudin-1.

**Figure S4 NMDS analysis of colonic and jejunal contents in different experimental groups.** The red ellipse is the result of bacterial aggregation in the colonic contents of CON, MOD and MXYS groups. The green ellipse is the result of bacterial aggregation in jejunal contents of CON, MOD and MXYS groups.

**Figure S5 Predicted functional profiling of bacterial communities in colon and jejunum.** (A) Colon content bacterial community function prediction pathway. (B) Jejunum content bacterial community function prediction pathway.

**Figure S6 Quality control and multivariate statistical analysis.** (A) PCA score plots of all analytical samples and and QC sample of colonic contents. (B) PCA score plots of all analytical samples and and QC sample of jejunal contents (C-D) Permutation test validation plots of the OPLS-DA model.

**Figure S7 The design of fecal bacteria transplantation experiment.**

**Table S1 Content of Saikosaponin (a, b2, b1, d) in three batches of MXYS**

| Batch | SS a (%) | SS b_2_ (%) | SS b_1_ (%) | SS d (%) | Total content (%) |
| --- | --- | --- | --- | --- | --- |
| 1 | 0.24 | 0.40 | 0.05 | 0.04 | 0.73 |
| 2 | 0.27 | 0.38 | 0.04 | 0.08 | 0.77 |
| 3 | 0.23 | 0.40 | 0.06 | 0.05 | 0.71 |

**Table S2 Content of paeoniflorin in three batches of MXYS**

| Batch | Paeoniflorin (%) |
| --- | --- |
| 1 | 0.654 |
| 2 | 0.662 |
| 3 | 0.608 |

**Table S3 Effects of behavioral and biochemical comparisons (‾x±s, n=6)**

| **Group** | **LPS** | **Effect Size** | **D-Lac** | **Effect Size** | **DAO** | **Effect Size** |
| --- | --- | --- | --- | --- | --- | --- |
| CON | 177.04±27.64 | CON vs. MOD  2.66  MOD vs. MXYS  2.58 | 475.71±45.55 | CON vs. MOD  3.09  MOD vs. MXYS  1.55 | 35.05±4.37 | CON vs. MOD  4.33  MOD vs. MXYS  3.20 |
| MOD | 251.26±23.03 |  | 653.24±58.46 |  | 52.21±2.67 |  |
| MXYS | 172.13±32.18 |  | 536.38±77.74 |  | 41.46±3.42 |  |
| **Group** | **Scocial interaction ratio** | **Effect Size** | **Body weight** | **Effect Size** | **Immobility time** | **Effect Size** |
| CON | 1.51±0.11 | CON vs. MOD  10.68  MOD vs. MXYS  6.83 | 21.42±0.35 | CON vs. MOD  2.05  MOD vs. MXYS  2.81 | 67.67±8.80 | CON vs. MOD  5.92  MOD vs. MXYS  6.40 |
| MOD | 0.44±0.06 |  | 20.12±0.74 |  | 125.21±8.95 |  |
| MXYS | 1.32±0.15 |  | 22.39±0.74 |  | 71.52±6.09 |  |
| **Group** | **Recognitive index** | **Effect Size** | **Sucrose preference ratio** | **Effect Size** | **The number of crossings** | **Effect Size** |
| CON | 86.03±3.68 | CON vs. MOD  7.64  MOD vs. MXYS  10.66 | 83.52±3.14 | CON vs. MOD  6.37  MOD vs. MXYS  10.67 | 105.83±3.89 | CON vs. MOD  8.78  MOD vs. MXYS  5.88 |
| MOD | 52.21±4.38 |  | 52.21±4.98 |  | 75.67±2.13 |  |
| MXYS | 92.26±2.09 |  | 93.81±2.68 |  | 89.67±2.21 |  |
| **Group** | **The time in center** | **Effect Size** | **The number of rearings** | **Effect Size** |  |  |
| CON | 1.37±0.19 | CON vs. MOD  1.95  MOD vs. MXYS  1.42 | 28.33±1.70 | CON vs. MOD  7.20  MOD vs. MXYS  9.51 |  |  |
| MOD | 2.80±0.93 |  | 15.83±1.46 |  |  |  |
| MXYS | 1.63±0.52 |  | 29.33±1.11 |  |  |  |

**Table S4 Bootstrap validation of key correlations (n=4)**

| **Correlation** | **r** | ***P* value** | **95% CI** | **Interpretation** |
| --- | --- | --- | --- | --- |
| Colon Claudin-1 vs. Social Interaction Ratio | 0.9679 | 1.3878E-9 | [0.888, 0.985] | Strong correlation |
| Colon Occludin vs. Social Interaction Ratio | 0.8480 | 0.002685 | [0.699, 0.958] | Strong correlation |
| Jejunal Claudin-1 vs. Social Interaction Ratio | 0.8484 | 0.000343 | [0.749, 0.977] | Strong correlation |
| Jejunal Occludin vs. Social Interaction Ratio | 0.8763 | 0.001316 | [0.606, 0.918] | Strong correlation |

**Table S5 Metabolic biomarkers in the colon content of mice (n = 8)**

| **No.** | **Metabolites** | **Formula** | **m/z** | **TR**  **（min）** | **VIP** | **MOD/CON**  **Trend** | **FDR** | **MXYS/MOD**  **Trend** | | **FDR** | **HMDB**  **ID** | **Ion mode** |
| --- | --- | --- | --- | --- | --- | --- | --- | --- | --- | --- | --- | --- |
| 1 | D-Proline | C5H9NO2 | 116.07 | 1.436 | 4.4774 | ↑^*^ | 0.028081 | ↓## | 0.009854 | | 0003411 | [M+H]^+^ |
| 2 | Cholic acid | C24H40O5 | 453.29 | 10.257 | 5.6617 | ↓^*^ | 0.021915 | ↑## | 0.001776 | | 0000619 | [M+FA-H]- |
| 3 | Hypoxanthine | C5H4N4O | 137.04 | 1.521 | 3.8512 | ↑^*^ | 0.001263 | ↓## | 0.016574 | | 0000157 | [M+H]^+^ |
| 4 | 3-Carboxyindole | C9H7NO2 | 162.05 | 6.434 | 6.4987 | ↓*** | 4.74E-05 | ↑### | 0.001413 | | 0003320 | [M+H]^+^ |
| 5 | Xanthine | C5H4N4O2 | 151.02 | 1.600 | 3.9438 | ↑** | 0.001499 | ↓## | 0.013028 | | 0000292 | [M-H]- |
| 6 | Guanine | C5H5N5O | 152.06 | 5.506 | 2.8104 | ↓** | 0.001263 | ↑# | 0.019711 | | 0000132 | [M+H]^+^ |
| 7 | 2-Aminobutyric acid | C4H9NO2 | 104.07 | 1.397 | 2.9962 | ↓*** | 0.007297 | ↑# | 0.0028033 | | 0000452 | [M+H]^+^ |
| 8 | Deoxycholic acid | C24H40O4 | 391.29 | 12.807 | 3.1076 | ↑*** | 0.018667 | ↓### | 0.030201 | | 0000626 | [M-H]- |
| 9 | Oleic acid | C18H34O2 | 283.26 | 20.465 | 2.4720 | ↓*** | 0.001263 | ↑## | 0.005722 | | 0000207 | [M+H]^+^ |
| 10 | N-Undecanoylglycine | C13H25NO3 | 266.17 | 9. 124 | 1.8981 | ↓*** | 0.008195 | ↑### | 0.001236 | | 0013286 | [M+Na]^+^ |
| 11 | N-Acetylproline | C7H11NO3 | 190. 11 | 1.489 | 1.9715 | ↑*** | 0.001263 | ↓## | 0.001557 | | 0094701 | [M+H+MeOH]^+^ |
| 12 | 4-Pyridoxic acid | C8H9NO4 | 184.06 | 3.018 | 1.1319 | ↑*** | 0.007745 | ↓### | 0.009854 | | 0000017 | [M+H]^+^ |
| 13 | Metyrosine | C10H13NO3 | 196.010 | 5.659 | 1.6172 | ↓** | 0.001488 | ↑## | 0.009854 | | 0014903 | [M+H]^+^ |
| 14 | Pyridoxal | C8H9NO3 | 168.07 | 1.450 | 1.6736 | ↑*** | 0.001612 | ↓### | 0.005173 | | 0001545 | [M+H]^+^ |
| 15 | Isovalerylglycine | C7H13NO3 | 160.10 | 1.48 | 1.2226 | ↑*** | 0.019411 | ↓### | 0.009854 | | 0000678 | [M+H]^+^ |
| 16 | Acetylcarnitine | C9H17NO4 | 204. 12 | 1.50 | 1.3870 | ↑** | 0.001612 | ↓# | 0.021004 | | 0000201 | [M+H]^+^ |
| 17 | 6-Hydroxypentadecane dioic acid | C15H28O5 | 287.19 | 6.64 | 1.0251 | ↑*** | 0.001263 | ↓### | 0.008195 | | 0031885 | [M-H]- |
| 18 | Uracil | C4H4N2O2 | 113.04 | 2.25 | 1.1063 | ↓** | 0.021375 | ↑# | 0.018667 | | 0000300 | [M+H]^+^ |
| 19 | Aspartate | C4H7NO4 | 133.100 | 5.692 | 1.0697 | ↓** | 0.001263 | ↑## | 0.005173 | | 0000191 | [M+H]^+^ |
| 20 | β-Alanine | C3H7NO2 | 90.055 | 1.304 | 1.8729 | ↓^*^ | 0.028081 | ↑# | 0.005173 | | 0000056 | [M+H]^+^ |
| 21 | L-Histidine | C6H9N3O2 | 309.133 | 7.474 | 1.2223 | ↓^*^ | 4.99E-04 | ↑# | 0.006436 | | 0000177 | [2M-H]- |

^*^*P* < 0.05, ^**^*P* < 0.01, ^***^*P* < 0.001, compared with the CON group; ^#^*P* < 0.05, ^##^*P* < 0.01, ^###^*P* < 0.001, compared with the MOD group.

**Table S6 Metabolic biomarkers in the jejunum content of mice (n = 8)**

| **No.** | **Metabolites** | **Formula** | **m/z** | **TR**  **（min）** | **VIP** | **MOD/CON**  **Trend** | **FDR** | **MXYS/MOD**  **Trend** | **FDR** | **HMDB**  **ID** | **Ion mode** |
| --- | --- | --- | --- | --- | --- | --- | --- | --- | --- | --- | --- |
| 1 | L-Norleucine | C6H13NO2 | 132.10 | 2.914 | 26.4166 | ↓^*^ | 0.025114 | ↑# | 0.014102 | 0001645 | [M+H]^+^ |
| 2 | Taurochenodeoxycholic acid | C26H45NO7S | 514.28 | 7.496 | 12.5371 | ↑### | 0.015605 | ↓### | 0.007376 | 0000949 | [M-H]- |
| 3 | L-Valine | C5H11NO2 | 118.09 | 2.156 | 5.82084 | ↓^*^ | 0.029715 | ↑# | 0.0098804 | 0000883 | [M+H]^+^ |
| 4 | Xanthine | C5H4N4O2 | 151.03 | 2.742 | 3.65406 | ↑^*^ | 0.001612 | ↓### | 0.010753 | 0000292 | [M-H]- |
| 5 | L-Pipecolic acid | C6H11NO2 | 147. 11 | 1.166 | 5.56402 | ↓^*^ | 0.00915 | ↑# | 0.014102 | 0000070 | [M+NH4]^+^ |
| 6 | Choline | C5H13NO | 104. 11 | 1.342 | 5.24766 | ↓^*^ | 0.029715 | ↑# | 0.039171 | 0000097 | [M+H]^+^ |
| 7 | Palmitic acid | C16H32O2 | 274.27 | 10.313 | 6.37547 | ↑*** | 0.014233 | ↓### | 0.003396 | 0000220 | [M+H]^+^ |
| 8 | 1-linoleoyl-2-hydroxy-sn-glycero-3-PC | C26H50NO7P | 520.34 | 15.296 | 6.76249 | ↑** | 0.0081452 | ↓## | 0.014102 | 0010386 | [M+H]^+^ |
| 9 | Glycerophosphocholine | C8H20NO6P | 258. 11 | 1.359 | 6.49202 | ↓^*^ | 0.021536 | ↑# | 0.014102 | 0000086 | [M+H]^+^ |
| 10 | Spermidine | C7H19N3 | 146.17 | 1.196 | 5.03931 | ↓*** | 0.0081452 | ↑### | 0.017418 | [0001257](http://www.hmdb.ca/metabolites/HMDB0001257) | [M+H]^+^ |
| 11 | Taurohyocholic acid | C_26_H45NO7S | 514.28 | 9.611 | 1.14534 | ↑* | 0.0081452 | ↓## | 1.54E-05 | 0000177 | [2M-H]- |
| 12 | 1-linoleoyl-sn-glycero-3-ph osphoethanolamine | C23H44NO7P | 478.29 | 15.193 | 2.50235 | ↑** | 0.015605 | ↓## | 0.019411 | 0011507 | [M+H]^+^ |
| 13 | Indole | C8H7N | 118.07 | 5.707 | 1.16762 | ↓^*^ | 0.0081452 | ↑# | 0.047344 | 0000738 | [M+H]^+^ |
| 14 | Pantothenic acid | C9H17NO5 | 218.10 | 5.814 | 1.8338 | ↓** | 0.0081452 | ↑# | 0.021375 | 0000210 | [M+H]^+^ |
| 15 | Retinol beta-glucuronide | C26H38O7 | 463.27 | 8.241 | 1.58814 | ↑*** | 0.0072966 | ↓### | 0.017363 | 0010340 | [M+H]^+^ |
| 16 | 1-Linolenoyl-sn-glycero-3- phosphocholine | C26H48NO7P | 518.32 | 14.225 | 1.43766 | ↑** | 0.025188 | ↓## | 0.021914 | 0010388 | [M+H]^+^ |
| 17 | 9, 12, 13-TriHOME | C18H34O5 | 329.23 | 9.346 | 1.14329 | ↓^*^ | 0.029715 | ↑# | 0.029468 | 0004708 | [M+H]^+^ |
| 18 | epsilon-(gamma-Glutamyl)-lysine | C11H21N3O5 | 276.156 | 3.869 | 1.1041 | ↑** | 0.029715 | ↓### | 0.018684 | 0003869 | [M+H]^+^ |

^*^*P* < 0.05, ^**^*P* < 0.01, ^***^*P* < 0.001, compared with the CON group; ^#^*P* < 0.05, ^##^*P* < 0.01, ^###^*P* < 0.001, compared with the MOD group.
